# C3d Complement Activation and Antibody Immunity in Convalescent COVID‐19: Insights From Adults and Children

**DOI:** 10.1155/jimr/7429749

**Published:** 2026-04-12

**Authors:** Ratna Wijaya, Jordan Stoddart, Anurag Adhikari, Arunasingam Abayasingam, Adam W. Bartlett, William Rawlinson, Michael M. Mina, Jeffrey J. Post, Bernard Hudson, Nicky Gilroy, Pam Konecny, Golo Ahlenstiel, Marianne Martinello, Andrew R. Lloyd, Nicodemus Tedla, Rowena A. Bull, Melanie R. Walker

**Affiliations:** ^1^ The Kirby Institute, University of New South Wales, Sydney, New South Wales, Australia, unsw.edu.au; ^2^ School of Biomedical Sciences, Faculty of Medicine, University of New South Wales, Sydney, New South Wales, Australia, unsw.edu.au; ^3^ Faculty of Medicine, University of Pelita Harapan, Tangerang, Indonesia; ^4^ Department of Infection and Immunology, Kathmandu Research Institute for Biological Sciences, Lalitpur, Nepal, kribs.org.np; ^5^ Sydney Children’s Hospital Randwick, Sydney, New South Wales, Australia, schn.health.nsw.gov.au; ^6^ Serology and Virology Division, Department of Microbiology, NSW Health Pathology, Prince of Wales Hospital, Sydney, New South Wales, Australia, princeofwalesprivatehospital.com.au; ^7^ Northern Beaches Hospital, Sydney, New South Wales, Australia, northernbeacheshospital.com.au; ^8^ Department of Infectious Diseases, Prince of Wales Hospital, Sydney, New South Wales, Australia, princeofwalesprivatehospital.com.au; ^9^ School of Clinical Medicine, University of New South Wales, Sydney, New South Wales, Australia, unsw.edu.au; ^10^ Royal North Shore Hospital, Sydney, New South Wales, Australia, nsw.gov.au; ^11^ Westmead Hospital, Sydney, New South Wales, Australia, nsw.gov.au; ^12^ St George Hospital, Sydney, New South Wales, Australia, nhs.uk; ^13^ Blacktown Clinical School, School of Medicine, Western Sydney University, Blacktown, New South Wales, Australia, westernsydney.edu.au; ^14^ Blacktown Hospital, Western Sydney Local Health District, Blacktown, New South Wales, Australia, nsw.gov.au; ^15^ Storr Liver Centre, The Westmead Institute for Medical Research, University of Sydney, Westmead, New South Wales, Australia, sydney.edu.au

## Abstract

Following severe acute respiratory syndrome coronavirus 2 (SARS‐CoV‐2) infection, the complement pathway becomes activated, generating the stable split product C3d. Although C3d can modulate B cell responses through complement receptor 2 (CR2) in experimental systems, it remains unclear how circulating C3d relates to antibody responses after SARS‐CoV‐2 infection in humans. Here, we examined plasma C3d levels and their associations with SARS‐CoV‐2–specific antibody responses and clinical characteristics in convalescent adults and children. In adults, older age, male sex, and a history of severe COVID‐19 were independently associated with higher C3d levels. C3d concentrations positively correlated with receptor‐binding domain (RBD)‐specific antibody‐dependent cellular phagocytosis (ADCP), neutralizing antibody (nAb) titer, and anti‐spike IgM, IgG, IgG1, and IgG3 levels. In children, C3d levels were significantly lower than in adults and correlated with anti‐spike and anti‐RBD IgG titers. These findings demonstrate that systemic complement activation, reflected by circulating C3d, is positively associated with SARS‐CoV‐2 antibody responses in both age groups. As this cross‐sectional study cannot determine causality, further mechanistic and longitudinal studies are needed to define the role, if any, of C3d in shaping adaptive immunity to SARS‐CoV‐2.

## 1. Introduction

The coronavirus disease 2019 (COVID‐19) pandemic, caused by severe acute respiratory syndrome coronavirus 2 (SARS‐CoV‐2), has posed a major burden to public health worldwide since its emergence in late 2019 [1]. The clinical outcome of COVID‐19 varies, ranging from asymptomatic to severe cases that can lead to death [1]. Although efficacious COVID‐19 vaccines are available, the continuous emergence of new SARS‐CoV‐2 variants and breakthrough infections indicates that an improved understanding of the host immune response towards SARS‐CoV‐2 is still needed. This may prove critical for further vaccine and therapeutic design for SARS‐CoV‐2 and other highly mutable pathogens.

Antibodies against SARS‐CoV‐2 that develop during infection are essential for viral clearance and protection against SARS‐CoV‐2 reinfection [2–5]. An effective antibody response toward SARS‐CoV‐2 generally relies on neutralizing antibody (nAb) development. NAbs bind to the SARS‐CoV‐2 spike protein, predominantly epitopes on the receptor‐binding domain (RBD), and prevent SARS‐CoV‐2 from infecting host cells via the angiotensin‐converting enzyme 2 (ACE2) receptors [6]. In addition to neutralization, Fc‐mediated functions such as antibody‐dependent cellular cytotoxicity (ADCC) and antibody‐dependent cellular phagocytosis (ADCP) also contribute to antiviral activity [7]. In SARS‐CoV‐2 infection, both ADCC and ADCP have been shown to correlate with antibody titer [8, 9], and ADCP is higher in subjects with severe COVID‐19 disease [9]. These Fc‐dependent pathways may be influenced by the inflammatory environment during infection, including complement activation [9].

Indeed, following SARS‐CoV‐2 infection, the complement system becomes activated [10]. The complement is a network of >30 plasma and membrane‐associated proteins activated through multiple pathways that converge at C3 cleavage [11]. C3 degradation ultimately generates C3d, a stable split product that can bind complement receptor 2 (CR2/CD21) on B cells and follicular dendritic cells (FDC). Although C3d–CR2 interactions have been shown in animal models to modulate B‐cell responses, the relevance of these mechanisms in human viral infection remains incompletely understood [11, 12]. Importantly, C3d detectable in plasma reflects systemic complement activation rather than tissue‐localized complement–antigen complexes and therefore does not directly indicate functional effects on B‐cell activity.

In SARS‐CoV‐2 infection, excessive and sustained complement activation is more prominent in individuals with severe disease and those with post‐COVID‐19 conditions [10, 13–16]. Increased levels of complement activation products, including C3d, and the deposition of C3d in the multiorgan system, have been associated with severe COVID‐19 disease outcomes and features, including endothelial injury and hypercoagulation [10, 17, 18]. Despite these observations, little is known about how circulating C3d levels vary in convalescent adults and children or how C3d relates to clinical characteristics and humoral immune profiles after infection. As both complement activation and antibody responses reflect disease severity and inflammation, their association may help explain differences in convalescent immunity.

In this study, we measured plasma C3d levels in adults and children who had recovered from COVID‐19 and examined their associations with age, prior disease severity, and SARS‐CoV‐2‐specific antibody responses. Our aim was to describe these relationships in convalescent individuals rather than infer mechanistic or causal effects of C3d on antibody immunity.

## 2. Materials and Methods

### 2.1. Study Subjects and Samples

This cross‐sectional study was conducted using EDTA plasma samples from unvaccinated COVID‐19 adults and children’s recovered cases from a prospective cohort (COSIN—Collection of COVID‐19 Outbreak Samples in New South Wales) [9, 19]. SARS‐CoV‐2 infection was confirmed by positive reverse transcriptase polymerase chain reaction (RT‐PCR) testing. Post‐acute infection follow‐up was scheduled at 1, 4, 8, 12, and 18 months after symptom onset or the date of RT‐PCR‐confirmed diagnosis (whichever came first). Here, blood samples from each subject were collected in EDTA vacutainers and separated by centrifugation. The serum was then collected and stored at −80°C until use. Standardized clinical data were also collected, which included disease severity stratified based on the National Institute of Health criteria as previously described [9, 19]. The samples were categorized as children (<18 years old) and adults (≥18 years old).

In this study, the median follow‐up plasma samples of convalescent COVID‐19 adults were collected at 8 weeks (IQR: 7–10), and, in children, at 7 weeks (IQR: 5–18) post the onset of symptoms or proven COVID‐19 disease by RT‐PCR positivity. Plasma samples of 25 subjects obtained from the Australian Red Cross collected before March 2019 were used in this study as negative controls.

### 2.2. Ethics Statement

Approval from the Human Research Ethics Committees of the Northern Sydney Local Health District and the University of New South Wales, NSW, Australia (ETH00520) was obtained, and the protocol was conducted along guidelines from the Declaration of Helsinki and International Conference on Harmonization Good Clinical Practice, as well as local regulatory requirements. Written and informed consent was obtained from all participants before the study procedures. All methods were performed in accordance with the relevant guidelines and regulations.

### 2.3. Quantification of Complement C3d Concentration by ELISA

The concentration of C3d was measured in EDTA plasma using commercially available ELISA kits (catalogue number A79152; Antibodies.com, Cambridge, United Kingdom) according to the manufacturer’s instructions. Sample wells were precoated with a monoclonal mouse antibody specific to human C3d. EDTA plasma samples were handled under conditions that minimize complement activation, including storage at ‐80°C for <5 years, minimal freeze/thaw cycles, and thawing at room temperature. In brief, EDTA plasma samples were diluted at 1:300 with the provided sample dilution buffer and then incubated at 37°C for 1.5 h. The sample wells were then washed twice, and 100 μL of biotinylated anti‐complement C3d detection antibody (rabbit polyclonal antibody recognizing distinct C3d epitopes) was added to each well and incubated at 37°C for 1 h. Following three washing steps to remove the unbound biotinylated detection antibody, 100 μL of horseradish peroxidase (HRP)‐streptavidin conjugate was added, and the plate was incubated at 37°C for 1 h. After a final wash, 90 μL TMB substrate solution was added and incubated at 37°C for 10 min. The reaction was stopped with 50 μL of stop solution, and the intensity of the color was measured using a microplate reader (CLARIOstar, BMG Labtech) at a 450 nm wavelength. The ELISA kit standard used in this study was provided by the manufacturer as a lyophilized powder consisting of the C3d region of human C3 (UniProt P01024; amino acids 1002–1303) expressed in *E. coli*. Preparation of the standard was performed according to the manufacturer’s instructions. Briefly, the standard was reconstituted with a sample dilution buffer to get a stock protein solution with a known concentration. Then, a serial dilution series of 1:2 was performed to obtain the standard curve. The ELISA standard was run in each plate to ensure the assay functionality and enable the quantification of the C3d concentration. Negative controls included 25 healthy controls (HCs) collected before March 2019, wavelength control, and blank samples. Data were then analyzed using a four‐parameter logistic (4‐PL) curve fit recommended by the manufacturer.

The C3d ELISA kit has no cross‐reactivity with mouse or rat samples and only cross‐reactivity with nonhuman primate samples according to the manufacturer. The dilution linearity across multiple sample dilutions was performed to measure the assay specificity, which showed recoveries of 80%–120%, indicating minimal interference from the sample matrix. All samples and standards for all assays were tested in duplicate. The intra‐assay and inter‐assay precision of this kit, measured in this study, represented as a percentage of the coefficient of variation (% CV), were <10% and <15%, respectively. These values reflect the good precision of the ELISA kit that was used in this study [20]. The manufacturer reports reference ranges of 3.4 –50 µg/mL for healthy human serum and 5.2–53 µg/mL for healthy human plasma. Each plate in our study included the kit standard curve, blank controls, and wavelength controls to ensure assay accuracy and consistency. Quality control samples at high, medium, and low concentrations are verified for each production batch to ensure lot‐to‐lot consistency.

### 2.4. Production of SARS‐CoV‐2 Recombinant Proteins

His‐tagged recombinant SARS‐CoV‐2 spike and RBD proteins were produced by transfecting Expi293‐Freestyle cells as previously described [21]. Briefly, 50 μg of each plasmid were transiently transfected into 1.5 × 10^8^ total (50 mL transfection). Expi293‐Freestyle cells, after which 160 μL of Expifectamin and 6 mL of OptiMEM‐I were added as per the manufacturer’s instructions. The following day, 300 μL of ExpiFectamine Enhancer 1 and 3 mL of ExpiFectamine Enhancer 2 were added to the cells. After 72 h, cell culture was collected and cleared of cellular debris. The His‐tagged protein was purified using a HisTrap HP column (GE Healthcare, Chicago, USA) and eluted with imidazole.

### 2.5. Detection of Anti‐SARS‐CoV‐2 Antibodies by Enzyme‐Linked Immunosorbent Assay (ELISA)

Antibodies (IgG, IgA, IgM, and IgG1−4) against SARS‐CoV‐2 spike or RBD proteins were measured by ELISA as described in our previous study [9], with the following modifications performed in children convalescence samples. In brief, Nunc MicroWell 96‐Well Microplates (ThermoFisher Scientific, Waltham, USA) were coated with 250 ng of recombinant SARS‐CoV‐2 RBD protein or 100ng of recombinant SARS‐CoV‐2 spike protein, diluted in phosphate buffered saline (PBS), and incubated overnight at 4°C. The plates were then washed with PBS containing 0.1% Tween‐20 (PBS‐T) (Sigma–Aldrich) and blocked with 5% skim milk in PBS‐T for 1 h at room temperature. After blocking, heat‐inactivated (56°C for 30 min) patient plasma samples, serially diluted in PBS‐T with 5% skim milk by a 3‐fold factor (1:20 to 1:393,660), were added to the plate in duplicate and incubated for 2 h at room temperature. Following another wash, anti‐human IgG conjugated with HRP (Jackson ImmunoResearch) diluted 1:3000 in PBS‐T with 5% skim milk was added to the plates and incubated for 1 h at room temperature. After a final wash, 3,3^′^, 5,5^′^‐tetramethylbenzidine (TMB) chromogen solution (ThermoFisher Scientific) was added to the plates, incubated for 15 min at room temperature, and then stopped with 1 M HCL. Optical density (OD) was measured at 450 nm using a CLARIOstar microplate reader (BMG LABTECH, Ortenberg, Germany). The background binding level to SARS‐CoV‐2 RBD was determined by taking the 1:20 dilution from 19 HCs and adding 2 standard deviations to their mean OD450. Endpoint titers (EPTs) were determined by interpolating the dilutions at which a sigmoidal curve intersected the background binding level.

### 2.6. ADCP Assay

The ADCP assay was performed as previously described [9]. Briefly, streptavidin and Alexa 488 fluorescent‐tagged 0.4 μm polyester microbeads (Spherotech, Lake Forest, USA) at 1.5 × 10^9^ beads/mL were mixed with biotinylated recombinant SARS‐CoV‐2 spike or RBD protein at 50 μg/mL in 1.5 mL tubes. The beads were incubated at 4°C for 16 h in a rotating chamber. Excess protein was removed by washing the beads twice with 1 mL Lipopolysaccharide‐minimized cold PBS (Gibco, Carlsbad, USA) and centrifuging at 2292 × *g* for 5 min.

Fifty microliter aliquots of the SARS‐CoV‐2 spike or RBD‐coated beads were opsonized for 2 h at 37°C with 10 μL of plasma from SARS‐CoV‐2‐ infected patients or HC donors. Next, 1 × 10^5^ THP‐1 cells (obtained from ATCC TIB‐202 [9]) were added to the plasma‐opsonized microbeads and mixed by gentle tapping. The mixture was then adjusted to 600 μL using RPMI 1640 containing 0.1% human serum and 100 mM HEPES and incubated at 37°C with 5% CO_2_.

Following a 2‐h incubation, the cells were washed with 1 mL of cold PBS containing 0.5% bovine serum albumin and 0.005% sodium azide (Sigma–Aldrich), gently centrifuged at 335 × *g* for 5 min at 4°C, fixed in 400 μL of 1% paraformaldehyde (ProSciTech, Kirwan, Australia), and stored at 4°C in the dark until processed with a BD FACSCanto II Flow Cytometer. The cells were analyzed with 2 × 10^4^ events recorded. Controls were created using cells incubated with spike or RBD‐coated beads opsonized with plasma from HCs. Phagocytosis scores (*p*‐scores) were calculated based on the proportion of cells that took up the opsonized beads (indicating the number of positive cells) and the mean fluorescence intensity (representing the average bead uptake by the positive cells).

### 2.7. SARS‐CoV‐2 Pseudovirus Neutralization Assay

The SARS‐CoV‐2 spike pseudovirus of the Wuhan‐Hu‐1 strain was generated in 293T cells using the Calphos transfection kit (Takara Bio) and expression plasmids containing SARS‐CoV‐2 spike, MLV gag/pol, and luciferase vectors, as described by us [9, 21]. The pseudovirus was harvested from the culture supernatants 48 h post‐transfection and concentrated 10‐fold using 100,000 MWCO Vivaspin centrifugal concentrators (Sartorius, Göttingen, Germany). For neutralization, the SARS‐CoV‐2 pseudovirus was preincubated for 1 h with heat‐inactivated (56°C for 30 min) patient serum before infecting 293 T‐ACE2 cells (kindly provided by A/Prof. J. Bloom). This was performed in quadruplicate via a 2‐h spinoculation at 800 × *g* in 96‐well white flat‐bottom plates (Sigma–Aldrich). Infected cells were then incubated at 37°C in a humidified incubator with 5% CO_2_ for 72 h and then lysed with a lysis buffer (Promega). Relative luminescence units in cell lysates were measured using a CLARIOstar microplate reader (BMG Labtech). The percentage neutralization of the spike pseudovirus was determined, and the 50% inhibitory concentration was calculated using a nonlinear regression model.

### 2.8. Statistical Analysis

GraphPad Prism software version 10.1.0 was used for data analysis and the preparation of the figures. Categorical variables were presented as numbers and percentages. Numerical variables were reported and presented in the figures as medians and interquartile ranges (IQRs). The Mann–Whitney *U* test was used for two independent group comparisons. Fisher’s exact or chi‐square tests were performed to compare categorical variables. Correlations between two continuous variables were calculated using a Spearman correlation test. A multivariable linear regression analysis was performed to identify the independent factors of demographic (age and gender) and clinical severity that affect the C3d levels. The variables of importance, including the disease severity, age, gender, days post infection (DPI), spike/RBD ADCP *p*‐score, anti‐spike/RBD EPT, anti‐spike/RBD antibody subtypes, and neutralization titer that may be associated with C3d levels, were ranked based on the change in *R* ^2^ relative to the full model of *R* ^2^ in multivariable linear regression analysis. The part correlation squared was calculated to identify the unique contribution of each variable in the full model of *R* ^2^ when the variables were removed from the regression equation. Furthermore, the covariance matrix was used to plot the intercorrelations between the independent variables related to C3d levels using the standardized *β* coefficient, ranging between ‐1 and 1, where a positive number indicates a positive relationship, 0 indicates no relationship, and a negative number indicates a negative relationship. Comorbidity and COVID‐19 therapy were not included as a covariate due to insufficient information. Results were considered statistically significant when the two‐sided *p*‐value was less than 0.05.

## 3. Results

### 3.1. Demographics and Clinical Data of the Study Cohort

The study included 62 convalescent COVID‐19 individuals, consisting of 45 adults and 17 children. The median age of the adult study group was 50 years (IQR: 31–67), and 23 (51%) were male. These individuals were classified into two clinical conditions: 9 (25%) had a history of severe COVID‐19 and 36 (75%) had a history of nonsevere COVID‐19, including 5 (14%) with asymptomatic disease, 15 (42%) with mild disease, and 16 (44%) with moderate disease. In our previous study [9], we tested nAb levels, spike and RBD antibody isotype/subtype (IgM, IgG, IgA, and IgG1−4) levels, and spike and RBD ADCP between individuals who had recovered from severe and nonsevere disease. These immunological parameters and demographic and clinical features of these convalescent adults are summarized in Table 1 and have been previously described [9].

**Table 1:** Characteristics of COVID‐19 convalescent adults.

Variable	Total (*n* = 45)	Severe (*n* = 9)	Nonsevere (*n* = 36)	*p*‐Value
Age, years	50 (31–67)	61 (39–78)	49 (26–66)	0.12
Sex				
Female	22 (49)	3 (33)	19 (58)	0.45
Male	23 (51)	6 (67)	17 (42)	
Time postinfection, weeks^a^	8 (7–10)	8 (7–13)	9 (7–10)	0.44
SARS‐CoV‐2 laboratory parameters				
Log_10_ nAb titer^b^	2.03 (1.57–2.38)	2.42 (2.03–3.03)	1.84 (1.49–2.22)	**0.006**
Log_10_ anti‐spike IgG titer	3.61 (3.20–4.34)	4.35 (3.97–4.49)	3.52 (3.15–3.97)	**0.001**
Anti‐spike, OD450 nm				—
IgM	0.34 (0.16–0.71)	1.09 (0.31–1.81)	0.30 (0.15–0.54)	**0.01**
IgA	0.07 (0.00–0.60)	0.32 (0.03–0.91)	0.01 (0.00–0.63)	0.24
IgG1	0.13 (0.07–0.40)	0.68 (0.29–0.98)	0.09 (0.07–0.20)	**0.0002**
IgG2	0.05 (0.05–0.06)	0.06 (0.05–0.07)	0.05 (0.05–0.06)	0.16
IgG3	0.48 (0.19–1.12)	1.24 (0.60–1.85)	0.38 (0.15–0.88)	**0.02**
IgG4	0.09 (0.07–0.11)	0.09 (0.08–0.11)	0.09 (0.07–0.11)	0.67
Spike phagocytic score, *p*‐score^c^	1169 (781.7–1539)	1669 (1196–1860)	1067 (638–1429)	**0.004**
Log_10_ anti‐RBD IgG titer	2.95 (2.66–3.56)	3.44 (2.88–4.11)	2.91 (2.53–3.43)	**0.03**
Anti‐RBD, OD450 nm	—	—	—	—
IgM	0.26 (0.14–0.49)	0.60 (0.29–1.05)	0.23 (0.13–0.41)	**0.005**
IgA	0.20 (0.08–0.38)	0.37 (0.20–0.44)	0.15 (0.04–0.34)	0.06
IgG1	0.08 (0.05–0.14)	0.16 (0.10–0.56)	0.06 (0.05–0.12)	**0.001**
IgG2	0.05 (0.04–0.06)	0.05 (0.05–0.06)	0.05 (0.04–0.06)	0.27
IgG3	0.23 (0.09–0.50)	0.48 (0.19–1.23)	0.16 (0.08–0.48)	**0.03**
IgG4	0.07 (0.06–0.09)	0.07 (0.06–0.08)	0.07 (0.06–0.09)	0.56
RBD phagocytic score, *p*‐score^c^	4.23 (1.38–6.82)	5.18 (4.58–8.02)	3.88 (1.16–6.80)	0.05

*Note*: Data are presented as median (interquartile range, IQR) or count (percentage). *p*‐Value was calculated using the Mann–Whitney *U* test for numerical variables and the chi‐square or the Fischer exact test for categorical variables. Bold values indicate statistical significance.

^a^Time postinfection was calculated from days after symptom onset or RT‐PCR positivity.

^b^The nAb titer was presented as the antibody titers that caused a 50% reduction of SARS‐CoV‐2 pseudovirus particle infectivity.

^c^Phagocytic scores (*p*‐score) were calculated as the proportion of THP‐1 cells that opsonized SARS‐CoV‐2 spike or RBD‐coated microbeads in the presence of patient plasma.

The median age of the children study group was 14 years (IQR: 9–15), with 5 (29%) being male. All COVID‐19 convalescent children experienced nonsevere manifestations of the COVID‐19 disease, with 13 (76%) recovering from mild disease, 2 (12%) recovering from asymptomatic disease, and 2 (12%) recovering from moderate disease. The demographic, clinical, and laboratory features of convalescent COVID‐19 children are listed in Table 2.

**Table 2:** The characteristics of COVID‐19 convalescent children.

Variable	Total (*n* = 17)
Age, years (median, IQR)	14 (9–15)
Sex, *n* (%)	
Female	12 (71)
Male	5 (29)
Time postinfection, weeks^a^ (median, IQR)	7 (5–18)
Clinical outcome, *n* (%)	
Asymptomatic	2 (12)
Mild	13 (76)
Moderate	2 (12)
SARS‐CoV‐2 laboratory parameters (median, IQR)	
Log_10_ anti‐spike IgG titer	3.17 (2.44–3.61)
Log_10_ anti‐RBD IgG titer	2.68 (2.06–3.31)

*Note:* Data are presented as median (interquartile range, IQR) or count (percentage). *p*‐Value was calculated using the Mann–Whitney *U* test for numerical variables and the chi‐square or the Fischer exact test for categorical variables.

^a^Time postinfection was calculated from days after symptom onset or RT‐PCR positivity.

### 3.2. C3d Levels Are Associated With Older Age, Male Gender, and Convalescent Adults Who Recovered From Severe COVID‐19 Disease

To characterize complement activation after acute SARS‐CoV‐2 infection, C3d levels among convalescent adult patients were quantified (*n* = 45) and compared with age‐ and sex‐matched HCs (*n* = 25). The median value of C3d concentration among convalescent COVID‐19 adults was 21.70μg/mL, which was significantly higher compared to HCs (median: 21.70 vs. 13.32 μg/mL, *p*  < 0.0001, Figure 1A). The median C3d concentration observed in our HCs (13.32 µg/mL) is consistent with values reported in previous studies using the same commercial ELISA kit in EDTA‐plasma from individuals without COVID‐19 [22, 23]. As a downstream and stable complement split product, C3d reflects cumulative complement activation but does not resolve the timing of activation events. Next, to determine whether C3d levels were associated with age, C3d levels were compared in those aged >50 and ≤ 50 years. Higher C3d concentrations were observed in older COVID‐19 convalescent individuals than in younger COVID‐19 convalescent individuals (median: 26.56 vs. 18.60 μg/mL, *p*  < 0.01, Figure 1B). When males and females were compared, males had greater C3d levels than females (median: 26.53 vs. 20.05μg/mL, *p*  < 0.05, Figure 1C). Furthermore, when compared by clinical condition, individuals who recovered from severe COVID‐19 disease exhibited higher C3d levels compared to those who experienced nonsevere COVID‐19 disease (median: 32.48 vs. 21.60 μg/mL, *p*  < 0.05, Figure 1D). Finally, we performed a multivariate regression analysis and found that older age (>50 years), male gender, and severe COVID‐19 disease were independently associated with increase in the C3d levels (*p*  < 0.05, Table 3).

Figure 1C3d levels among convalescent COVID‐19 adults. (A) C3d levels were compared between healthy controls (HCs) and convalescent COVID‐19 subjects. C3d levels were also analyzed by demographic ((B) age and (C) sex) and (D) disease severity among COVID‐19 subjects. Statistical analysis was performed using the Mann–Whitney *U* test to compare two independent groups,  ^∗^ *p* < 0.05,  ^∗∗^ *p* < 0.01,  ^∗∗∗∗^ *p* < 0.0001.

**Figure:**
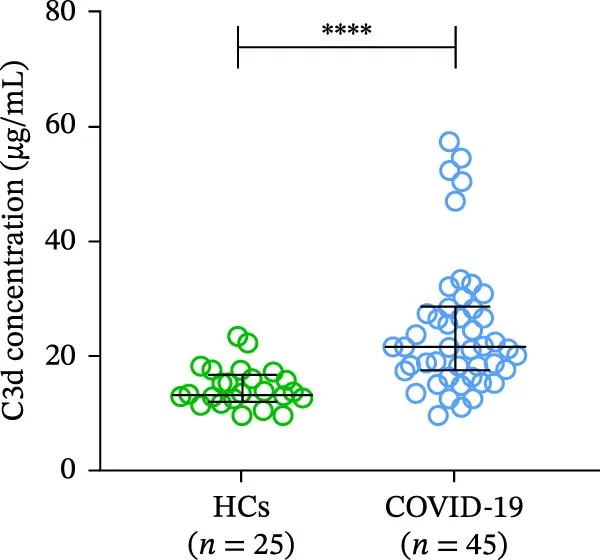
(A)

**Figure:**
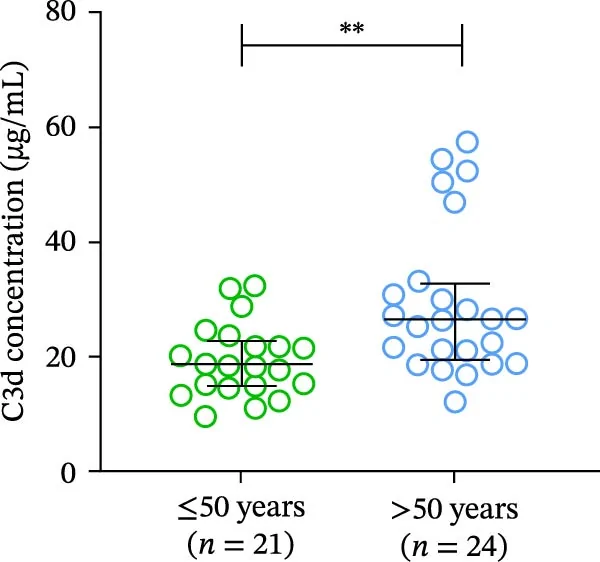
(B)

**Figure:**
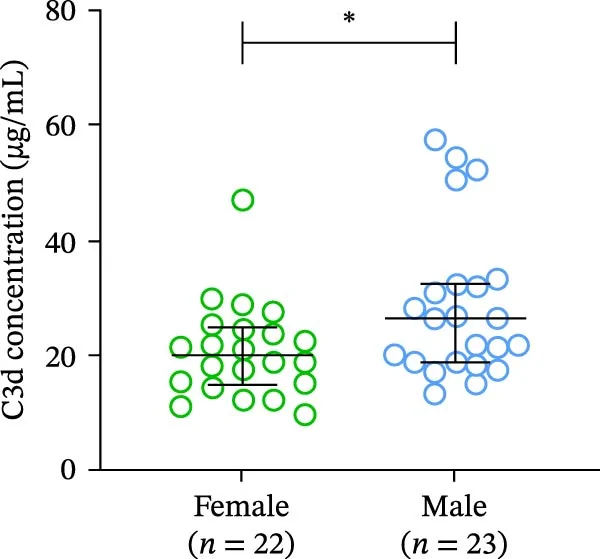
(C)

**Figure:**
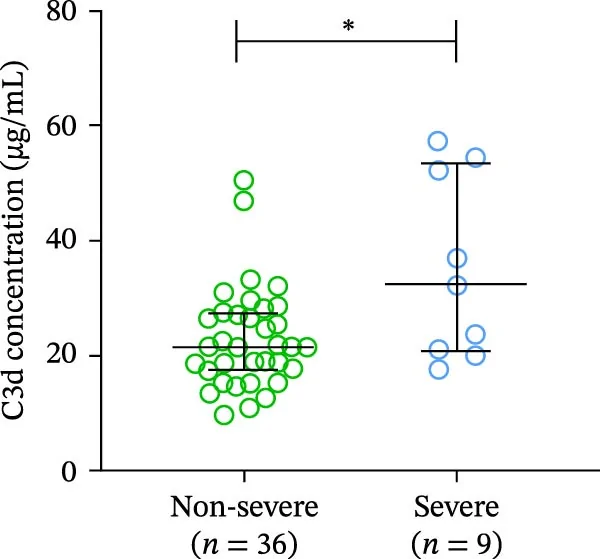
(D)

**Table 3:** Multivariable linear regression analysis of factors related to C3d levels in convalescent COVID‐19 adults.

Variable	Beta (95% CI)	*p*‐Value
Age (>50 vs. ≤50 years)	10.43 (4.12–16.73)	0.001
Sex (male vs. female)	7.86 (1.55–14.18)	0.015
COVID‐19 disease outcome (severe vs. nonsevere)	8.54 (0.59–14.18)	0.035

Abbreviation: CI, confidence interval.

### 3.3. C3d Levels and Antibody‐Mediated Immunity in Convalescent COVID‐19 Adults

To examine the relationship between C3d levels and antibody‐mediated immunity in post‐acute SARS‐CoV‐2 infection, we assessed the association between C3d levels and ADCP responses, nAb response, anti‐spike/RBD IgA, IgM, IgG levels, and IgG subtype (IgG1−4) antibody levels among COVID‐19 convalescent adults. A significant positive correlation was found between C3d levels and nAb titers (*r* = 0.435, *p* = 0.002, Figure 2A), anti‐spike IgG (*r* = 0.336, *p* = 0.013, Figure 2B), anti‐spike IgM (*r* = 0.416, *p* = 0.004, Figure 2C), anti‐spike IgG1 antibody levels (*r* = 0.374, *p* = 0.011, Figure 2D), IgG3 antibody levels (*r* = 0.331, *p* = 0.026, Figure 2E), and RBD ADCP *p*‐score (*r* = 0.339, *p* = 0.022, Figure 2F). No significant correlation was observed between anti‐RBD IgA, IgM, IgG levels, and IgG subtype (IgG1−4), anti‐spike IgA, IgG2, IgG4, or spike ADCP *p*‐score with C3d concentration (Figure S1). These data show that higher plasma C3d levels are observed in individuals who also have higher SARS‐CoV‐2 nAb titers and selected spike‐specific antibody isotypes/subclasses and Fc‐mediated functions at the convalescent time point. These findings demonstrate coordinated associations between systemic complement activation and specific features of the SARS‐CoV‐2 antibody response during convalescence, while not establishing causal or directional relationships.

Figure 2The association of C3d levels with antibody‐mediated immunity among convalescent COVID‐19 adults. The association of C3d levels with (A) nAbs against spike SARS‐CoV‐2 protein, (B–E) antibody isotype and IgG subtype immune response against spike SARS‐CoV‐2 protein, and (F) antibody‐mediated phagocytosis activities (*p*‐score) against spike RBD SARS‐CoV‐2 protein. Number of samples for analysis, *n* = 45. Statistical analysis was performed using the Spearman rank correlation test to evaluate correlations.

**Figure:**
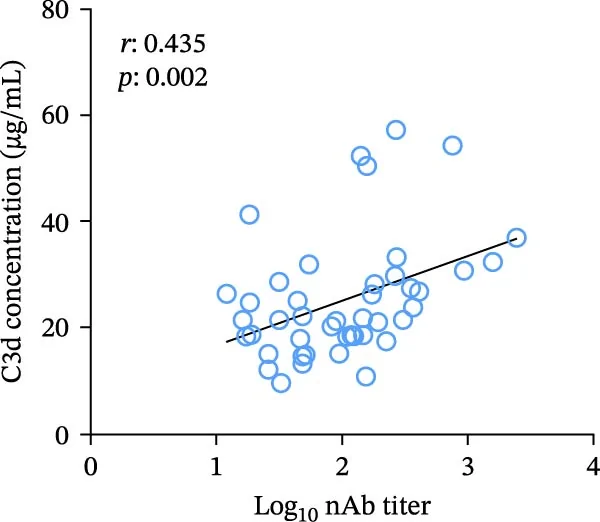
(A)

**Figure:**
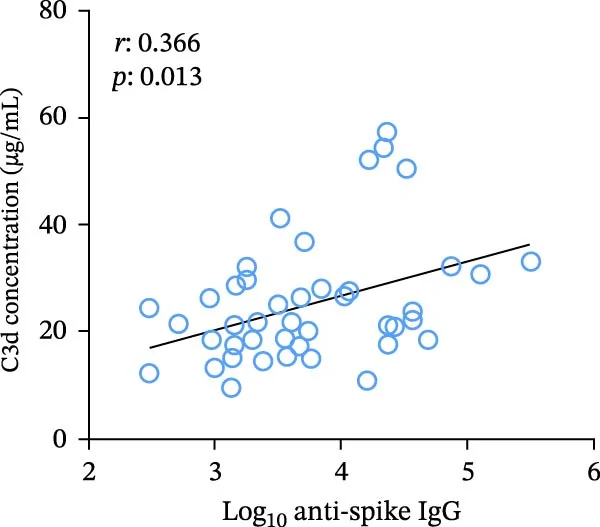
(B)

**Figure:**
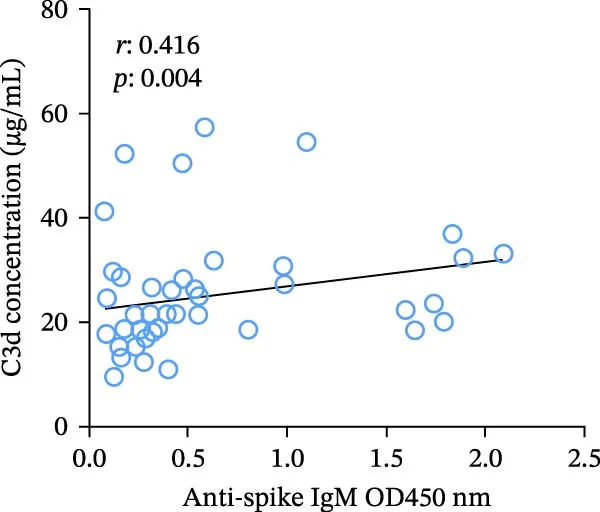
(C)

**Figure:**
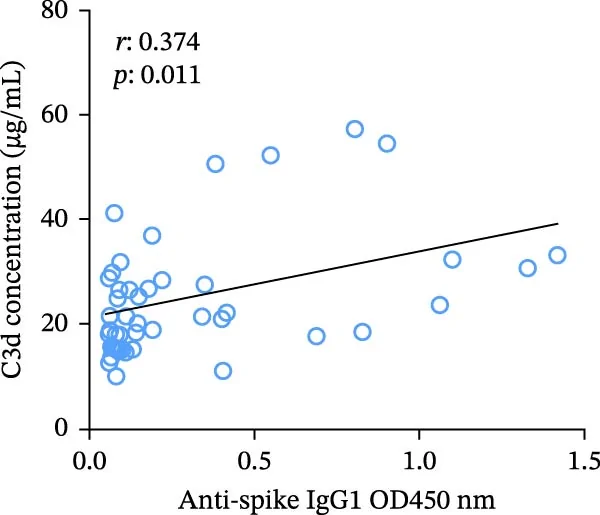
(D)

**Figure:**
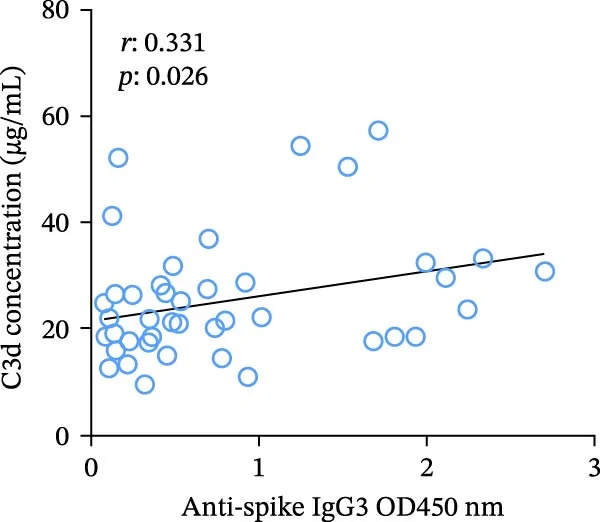
(E)

**Figure:**
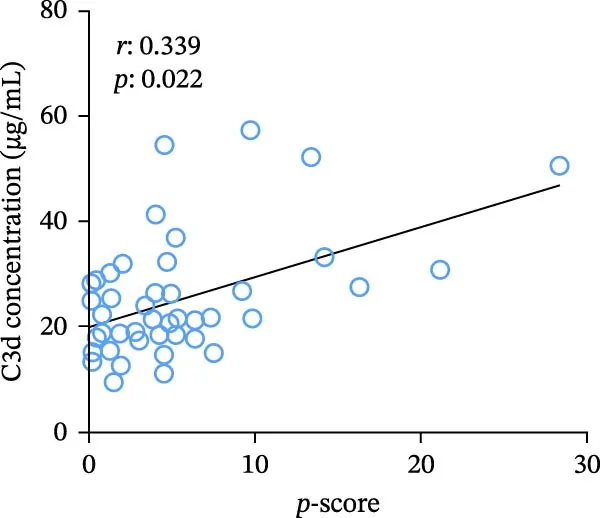
(F)

### 3.4. Ranking Variables of Importance Associated With C3d Levels

**Figure 3:**
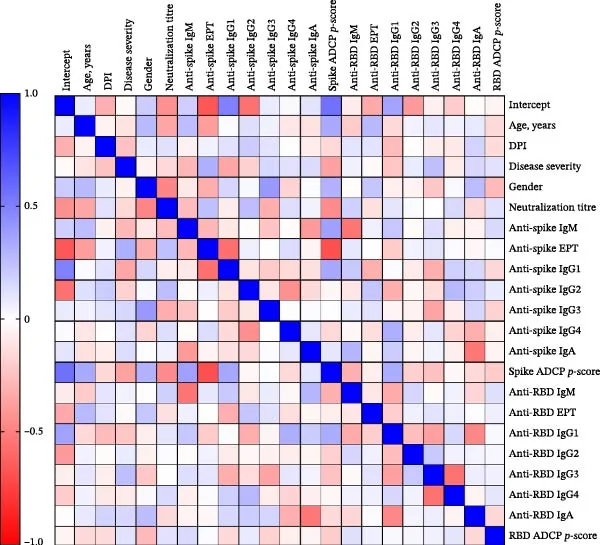
The covariance matrix of clinical and laboratory variables that related to C3d levels among convalescent COVID‐19 adults. Covariance matrix analysis was performed to show the intercorrelations among clinical and laboratory parameters related to C3d levels using the standardized *β* coefficient covariance scale (range −1 to 1), where a positive number (blue color) indicates a positive relationship, 0 (white color) indicates no relationship, and a negative number (red color) indicates a negative relationship.

Multiple linear regression analysis was performed to investigate the relationship of clinical and experimental variables with C3d levels in convalescent COVID‐19 adults. Overall, this resulted in a significant model with *p*‐value = 0.009 and *R* ^2^ = 0.719. To identify the most important variables in regression models that were related to C3d levels, we measured the change in *R* ^2^ when each variable was removed from the full model. Age was the most important variable that contributed to the C3d level (change in *R* ^2^ = 0.0915) (Table 4), followed by gender (change in *R* ^2^ = 0.0807) and history of disease severity (change in *R* ^2^ = 0.0610), while the rest of the variables are listed in descending order of importance in Table 4. A covariance matrix was plotted to determine the associative relationship between the clinical and the laboratory variables related to the C3d levels. We observed that age and disease severity were positively correlated with most independent variables (Figure 3). In addition, the antibody isotypes and subtypes, anti‐RBD IgM, IgG, and IgG3, were related to most independent variables and showed a positive trend towards C3d levels, indicating that C3d levels tend to be higher in individuals who also have higher levels of several SARS‐CoV‐2–specific antibody measures. However, these analyses do not distinguish the specific complement pathways contributing to C3d generation (Figure 3).

**Table 4:** Multiple regression analysis of ranking of variables of importance for C3d.

Variable	Change in *R* ^2^
Age, years	0.0915
Gender	0.0807
Disease severity	0.0610
DPI	0.0424
Anti‐RBD IgG3	0.0411
Anti‐spike IgG4	0.0386
Anti‐spike IgG3	0.0365
RBD ADCP *p*‐score	0.0356
Anti‐spike IgM	0.0299
Anti‐spike IgG2	0.0267
Neutralization titer	0.0193
Anti‐RBD IgG1	0.0130
Anti‐spike IgG1	0.0111
Anti‐RBD IgG4	0.0088
Anti‐spike EPT	0.0039
Anti‐RBD IgA	0.0012
Anti‐RBD IgM	0.0004
Anti‐spike IgA	0.0001
Anti‐RBD EPT	0.0001
Anti‐RBD IgG2	0.0000
Spike ADCP *p*‐score	0.0000

Abbreviations: DPI, days post infection; EPT, end point titer.

### 3.5. C3d Levels in Convalescent COVID‐19 Children

Given that activation of the complement system has been associated with severe COVID‐19, post‐COVID‐19 conditions, and age [15, 16, 24], we compared C3d levels between children and adults to gain a better understanding of the relationship between age, C3d levels, and COVID‐19 disease outcome in children. First, C3d levels between adults and children were compared to determine whether C3d levels were elevated in either group. Lower C3d levels in convalescent children were observed compared to convalescent adults with history of nonsevere COVID‐19 disease (median: 16.12 vs. 21.60 μg/mL, *p*  < 0.001; Figure 4A) and severe COVID‐19 disease (median: 16.21 vs. 32.48 μg/mL, *p*  < 0.0001; Figure 4A). No significant difference was observed in C3d levels according to children’s age groups (<12 and ≥12;Figure 4B) and gender variables (Figure 4C). Next, we assessed anti‐spike IgG, anti‐RBD IgG, and C3d levels in convalescent COVID‐19 children to determine the association between complement activation and antibody responses. As shown in Figure 4D, C3d levels positively correlated with anti‐spike IgG (*r* = 0.593, *p* = 0.013) and anti‐RBD IgG (*r* = 0.490, *p* = 0.047). Overall, while C3d levels are lower in children than adults, circulating C3d remains positively associated with anti‐spike and anti‐RBD IgG levels in children, mirroring the pattern observed in adults and suggesting coordinated regulation of complement activation and humoral immunity across age groups; however, the directionality and mechanistic basis of this relationship cannot be determined from these cross‐sectional data.

Figure 4C3d levels analysis among convalescent COVID‐19 children. (A) Differences in C3d levels were observed between convalescent COVID‐19 children and adults. The C3d levels were analyzed by demographic (B) age, (C) sex, and (D) their correlation with anti‐spike IgG and anti‐RBD IgG antibody levels. Number of samples for analysis, *n* = 17. Statistical analysis was performed using a Mann–Whitney *U* test to compare two independent groups and the Spearman rank correlation test to evaluate correlations,  ^∗∗∗^ *p*  < 0.001,  ^∗∗∗∗^ *p*  < 0.0001.

**Figure:**
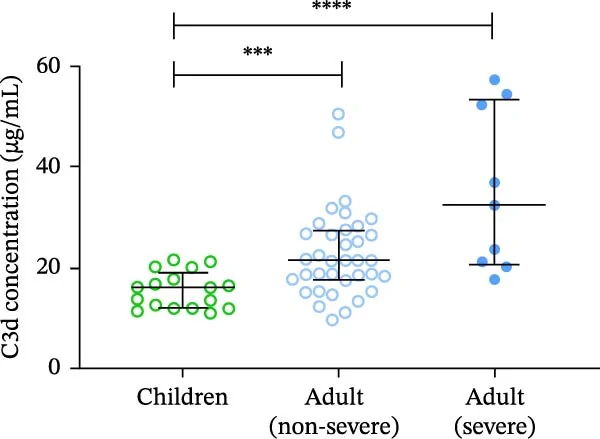
(A)

**Figure:**
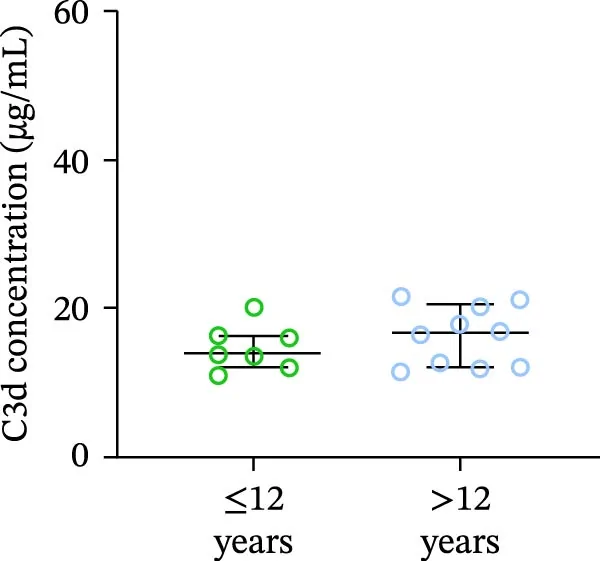
(B)

**Figure:**
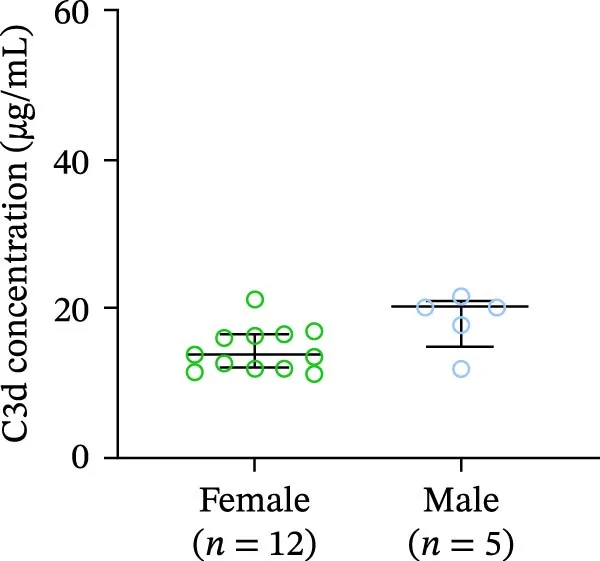
(C)

**Figure:**
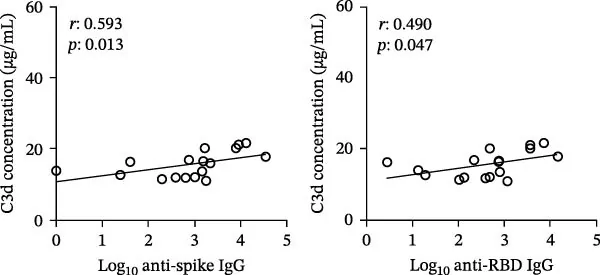
(D)

## 4. Discussion

During SARS‐CoV‐2 infection, the host complement system has been shown to play an antiviral role and has been associated with disease severity and post‐COVID‐19 conditions [13–16]. In addition, in human and mice studies, the complement system can modulate the humoral immune responses, primarily through human complement receptor type 2 (CR2 and CD21) which is expressed on B cells and FDC and binds to complement cleavage products C3d with high affinity [11, 12]. While these mechanisms are well described experimentally, their relevance to circulating C3d levels in human viral infection remains uncertain. Here, we measure C3d and its association with antibody‐mediated immunity in convalescent COVID‐19 adults and children. We observed an increase in the complement activation product C3d following COVID‐19 disease, which was associated with older age, male gender, and recovery from severe COVID‐19. Furthermore, C3d levels positively correlated with RBD‐specific ADCP, nAb titers, anti‐spike IgM, IgG, IgG1, and IgG3 antibody levels in convalescent COVID‐19 adults and anti‐spike IgG and anti‐RBD IgG in children. These associations show that higher systemic complement activation tends to occur in individuals with stronger measurable antibody responses. Although the cross‐sectional design means we cannot infer directionality, these associations are consistent with either direct links between complement activation and humoral immunity, shared upstream drivers such as viral burden or inflammation, or a combination of these mechanisms.

Previous studies have shown that complement component concentration and activity are influenced by age and sex [24, 25], with C3 levels and complement activation through classical, alternative, or lectin pathways being higher in males and significantly correlated with age [24–27]. In this study, age and male sex were significantly associated with higher C3d levels. This pattern aligns with known demographic differences in complement biology, and multiple regression analysis identified age as the strongest predictor. However, our covariance analysis and individual‐level data also highlight substantial differences between subjects, not all older or male individuals had elevated C3d, and some individuals with nonsevere disease showed levels overlapping those with severe disease. This variability suggests that complement activation is shaped by multiple interacting host and disease factors beyond age and sex alone.

We also found higher C3d levels in individuals with prior severe COVID‐19, supporting previous reports that complement activation is more pronounced in severe disease [14, 16, 17]. This finding aligns with previous studies that showed the prolonged elevation of complement activation markers in convalescent individuals with a history of severe COVID‐19 disease [28, 29]. C3d, a stable split product of C3, reflects cumulative complement activation across pathways [10, 30, 31]. As a downstream fragment with a prolonged half‐life, circulating C3d represents integrated rather than acute complement activity and cannot distinguish between recent and earlier activation. Circulating levels may also be affected by renal clearance. Among the individuals with the highest C3d concentrations, two had documented chronic kidney disease; however, detailed renal function measurements were not available, and no cases were recorded as dialysis‐dependent or end‐stage renal disease. Elevated C3d levels were also observed in individuals without documented renal disease. These findings suggest that renal insufficiency alone is unlikely to account for the observed associations, although its potential contribution cannot be excluded. Accordingly, our findings are interpreted as associations between complement activation and antibody responses, not as evidence of causality. The sustained elevation observed in some convalescent individuals may relate to this longer half‐life and to persistent vascular or immune dysregulation following infection [32–37]. Notably, individuals with severe acute disease did not uniformly show high C3d levels in convalescence, indicating variability in post‐acute complement activation.

In adults, C3d correlated with nAb titers and spike‐specific IgM and IgG, including IgG1 and IgG3, which are key mediators of neutralization and Fc effector functions [38–40]. These observations suggest that complement activation and humoral profiles covary after infection but do not establish causality. Several interpretations remain possible: complement activation may contribute to shaping antibody responses, both may be independently driven by viral burden and inflammation, or host factors may influence both pathways. Given that IgG1 and IgG3 responses depend on T helper cell support [41], our findings are consistent with, but do not prove, a connection to broader germinal center activity.

We also observed a correlation between C3d levels and RBD‐specific ADCP but no correlation with spike‐specific ADCP activity. RBD‐focused responses may more closely track with overall inflammatory activity, but our data cannot determine whether C3d directly contributes to ADCP. Additionally, the low expression of CD11b, a receptor component of CR3, on THP‐1 cells may limit the ability to detect complement‐mediated enhancement in this model [42].

In children, C3d correlated with anti‐spike and anti‐RBD IgG, mirroring adult patterns but at lower levels. As in adults, these findings should be interpreted as associations rather than directional effects. Convalescent children displayed significantly lower C3d levels than adults, consistent with age‐related differences in complement activation [24, 26], and potentially contributing to lower risk of severe disease [43–45]. Despite lower complement activation, children mount robust spike‐ and RBD‐specific IgG responses, supporting a distinct immunological profile relative to adults.

Several limitations should be acknowledged. The sample size was modest, especially in children. Pre‐infection C3d levels were not available, as is expected in a pandemic setting. Therefore, we included pre‐2019 HCs to understand baseline levels. The cross‐sectional design limits causal inference or assessment of antibody durability. We evaluated antibodies only against the Wuhan spike and RBD and did not assess T cell–mediated immunity, which has been linked to mild disease [46]. As this study does not include cellular immunity parameters, it provides only partial information regarding the relationship of C3d levels with COVID‐19 immune protection. Plasma C3d may not reflect tissue‐level complement activity, and unmeasured clinical factors such as comorbidities, treatments, BMI, smoking, and C3 variants may contribute to variability.

In conclusion, we identified associations between C3d levels, demographic factors, disease severity, and SARS‐CoV‐2–specific antibody responses in convalescent adults and children. These findings highlight variation in complement activation and its association with humoral immunity, providing a basis for future mechanistic and longitudinal investigations. Further work is required to determine whether C3d plays any causal role in shaping adaptive immunity or may serve as a biomarker or therapeutic target.

## Funding

Research reported in this publication was supported by Snow Medical Foundation as an investigator‐initiated study. Ratna Wijaya receives support from the APEC‐Australia Women in Research Fellowship. Open access publishing facilitated by University of New South Wales, as part of the Wiley ‐ University of New South Wales agreement via the Council of Australasian University Librarians.

## Conflicts of Interest

The authors declare no conflicts of interest.

## Supporting Information

Additional supporting information can be found online in the Supporting Information section.

## Supporting information


**Supporting Information** Supporting Figure S1. The association of C3d levels with antibody‐mediated immunity among convalescent COVID‐19 adults. The association of C3d levels with (A) antibody immune response against spike SARS‐CoV‐2 protein, (B) antibody immune response against spike RBD SARS‐CoV‐2 protein. Statistical analysis was performed using the Spearman rank correlation test to evaluate correlations.

## Data Availability

The data that support the findings of this study are available from the corresponding author upon reasonable request.
