# Supplementary material for: C3d Complement Activation and Antibody Immunity in Convalescent COVID‐19: Insights From Adults and Children
Source: J Immunol Res. 2026 Apr 12;2026:7429749. doi: 10.1155/jimr/7429749 (PMC13581050; doi:10.1155/jimr/7429749)
Supplement: Supplementary file 1 [file JIMR-2026-7429749-s001.pdf]

Supplementary information

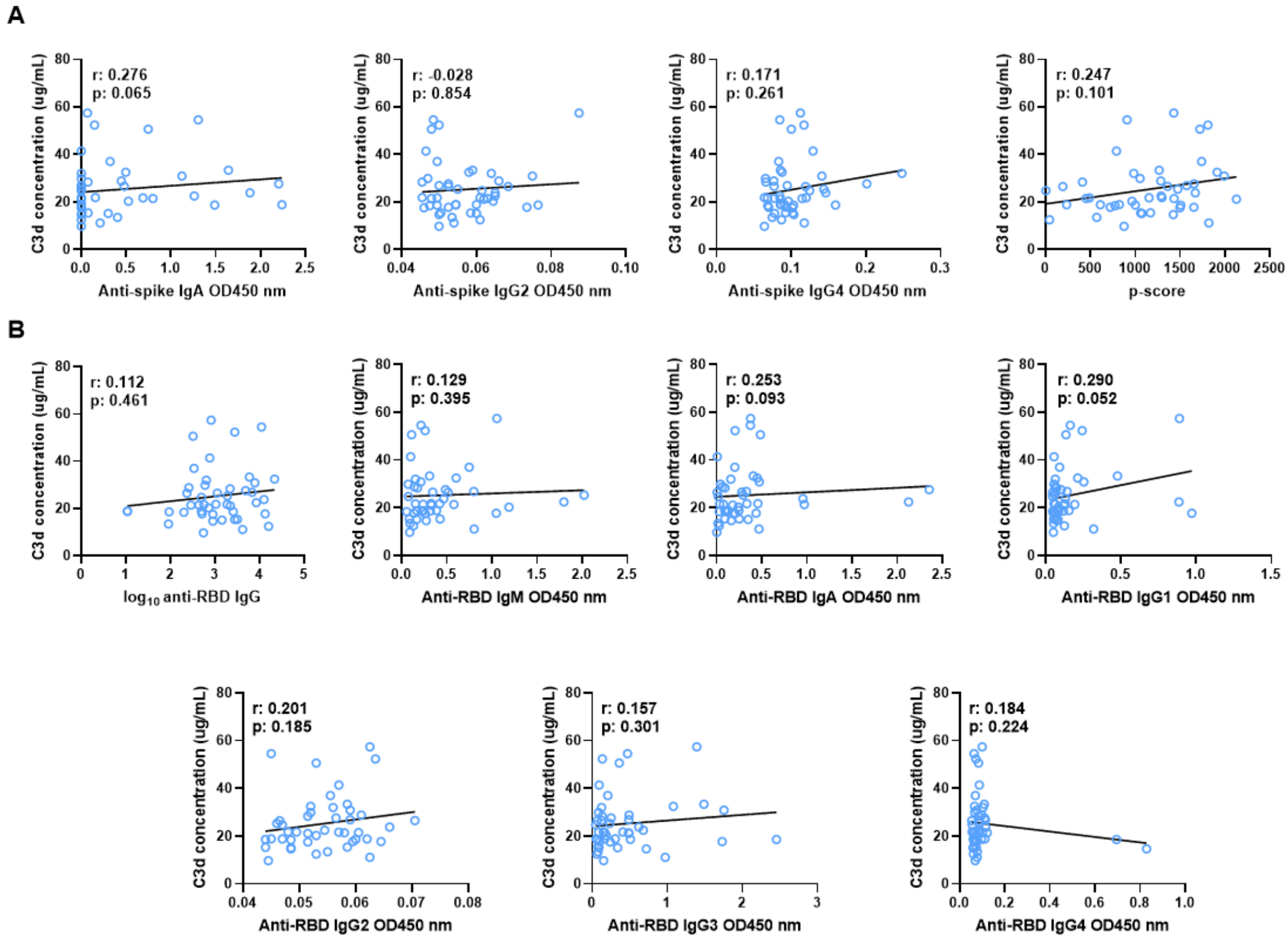

Supplementary Figure 1. **The association of C3d levels with antibody-mediated immunity among convalescent COVID-19 adults**.

The association of C3d levels with (A) antibody immune response against spike SARS-CoV-2 protein, (B) antibody immune response against spike RBD SARS-CoV-2 protein. Statistical analysis was performed using the Spearman rank correlation test to evaluate correlations.
